# Genetic analysis of low-grade adenosquamous carcinoma of the breast progressing to high-grade metaplastic carcinoma

**DOI:** 10.1007/s10549-023-07078-9

**Published:** 2023-08-31

**Authors:** Kae Kawachi, Xiaoyan Tang, Rika Kasajima, Takashi Yamanaka, Eigo Shimizu, Kotoe Katayama, Rui Yamaguchi, Kazuaki Yokoyama, Kiyoshi Yamaguchi, Yoichi Furukawa, Satoru Miyano, Seiya Imoto, Emi Yoshioka, Kota Washimi, Yoichiro Okubo, Shinya Sato, Tomoyuki Yokose, Yohei Miyagi

**Affiliations:** 1 Department of Pathology, Kanagawa Cancer Center, 2-3-2 Nakao, Aasahi-ku, Yokohama, Japan; 2 Department of Pathology, The Jikei University School of Medicine, 3-25-8 Nishishinbashi, Minato-ku, Tokyo, Japan; 3 Department of Pathology, Nihon University Hospital, 1-6 Kandasurugadai, Chiyoda-ku, Tokyo, Japan; 4 Molecular Pathology and Genetics Division, Kanagawa Cancer Center Research Institute, 2-3-2 Nakao, Aasahi-ku, Yokohama, Japan; 5 Division of Health Medical Intelligence, Human Genome Center, Institute of Medical Science, The University of Tokyo, 4-6-1 Shirokanedai, Minato-ku, Tokyo, Japan; 6 Department of Breast and Endocrine Surgery, Kanagawa Cancer Center, 2-3-2 Nakao, Aasahi-ku, Yokohama, Japan; 7 Division of Cancer Systems Biology, Aichi Cancer Center Research Institute, 1-1 Kanokoden, Chikusa-ku, Nagoya, Japan; 8 Division of Cancer Informatics, Nagoya University Graduate School of Medicine, 65 Tsurumai-Cho, Showa-ku, Nagoya, Japan; 9 Department of Hematology/Oncology, Research Hospital, Institute of Medical Science, The University of Tokyo, Tokyo, Japan; 10 Division of Clinical Genome Research, Institute of Medical Science, The University of Tokyo, 4-6-1 Shirokanedai, Minato-ku, Tokyo, Japan; 11 Department of Integrated Data Science, Medical and Dental Data Science Center, Tokyo Medical and Dental University, 2-3-10 Kandasurugadai, Chiyoda-ku, Tokyo, Japan

**Keywords:** Low-grade adenosquamous carcinoma, High-grade progression, Whole-genome sequencing, Metaplastic carcinoma, Breast cancer

## Abstract

**Purpose:**

Low-grade adenosquamous carcinoma (LGASC) is a rare type of metaplastic carcinoma of the breast (MBC) with an indolent clinical course. A few LGASC cases with high-grade transformation have been reported; however, the genetics underlying malignant progression of LGASC remain unclear.

**Methods:**

We performed whole-genome sequencing analysis on five MBCs from four patients, including one case with matching primary LGASC and a lymph node metastatic tumor consisting of high-grade MBC with a predominant metaplastic squamous cell carcinoma component (MSC) that progressed from LGASC and three cases of independent de novo MSC.

**Results:**

Unlike de novo MSC, LGASC and its associated MSC showed no *TP53* mutation and tended to contain fewer structural variants than de novo MSC. Both LGASC and its associated MSC harbored the common *GNAS* c.C2530T:p.Arg844Cys mutation, which was more frequently detected in the cancer cell fraction of MSC. MSC associated with LGASC showed additional pathogenic deletions of multiple tumor-suppressor genes, such as *KMT2D* and *BTG1*. Copy number analysis revealed potential 18q loss of heterozygosity in both LGASC and associated MSC. The frequency of *SMAD4::DCC* fusion due to deletions increased with progression to MSC; however, chimeric proteins were not detected. SMAD4 protein expression was already decreased at the LGASC stage due to unknown mechanisms.

**Conclusion:**

Not only LGASC but also its associated high-grade MBC may be genetically different from de novo high-grade MBC. Progression from LGASC to high-grade MBC may involve the concentration of driver mutations caused by clonal selection and inactivation of tumor-suppressor genes.

**Supplementary Information:**

The online version contains supplementary material available at 10.1007/s10549-023-07078-9.

## Introduction

Metaplastic carcinoma of the breast (MBC) is a subtype of invasive breast carcinoma with differentiation to squamous epithelium and mesenchymal components; MBC accounts for 0.2–1% of all invasive breast carcinomas [1]. Most MBCs are triple-negative breast carcinomas. MBCs usually have poor outcomes and low response rates to conventional chemotherapy; however, obvious prognostic or predictive factors of therapeutic response remain unclear [1]. Overall, MBCs are clinically, morphologically, and genetically heterogeneous tumors; histologically low-grade tumors with favorable prognosis, such as low-grade adenosquamous carcinoma (LGASC) and fibromatosis-like carcinoma, are rarely recognized.

LGASC is characterized by well-developed glandular and tubular formation admixed with solid nests of squamous cells in a spindle cell background [1, 2]. Diagnosis based on core biopsy is challenging due to the difficulty in distinguishing between neoplastic and nonneoplastic glands. Although LGASC has been suggested to be related to some benign breast proliferative lesions [3–6], its pathogenesis remains unclear. The relatively high recurrence rate after excision biopsy suggests local aggressiveness, yet metastasis is rare, with only one case each of lymph node and distant metastasis documented [6, 7]. Furthermore, few cases with transition to high-grade MBC have been reported to date [8–12].

Molecular studies have clarified that high-grade MBC frequently harbors *TP53* mutations, as observed in triple-negative invasive ductal carcinomas of no special type [13–15]. One study reported more frequent mutations in genes associated with the activation of the PI3K-AKT and Wnt pathways in MBC than triple-negative invasive ductal carcinoma of no special type [13]. *TP53* mutation in LGASC has not been reported, whereas approximately half of cases harbor *PIK3CA* mutation, with most cases showing genetic alterations involving in the PI3K-AKT pathway [4, 8, 16]. Nevertheless, the characteristics of the genetic alterations associated with the transition of LGASC to high-grade MBC have not been elucidated.

Therefore, this study was designed to explore the features of genetic alterations in LGASC progressing to high-grade MBC compared to de novo high-grade MBC.

## Materials and methods

### Case selection and clinicopathologic characteristics

This study included four patients with breast tumors who underwent resection at Kanagawa Cancer Center and were diagnosed with MBC. Case 1 diagnosis was primary LGASC that progressed to high-grade MBC with a predominant metaplastic squamous cell carcinoma component (MSC). Diagnoses in Cases 2, 3, and 4 were de novo MSC, and archival specimens were retrieved from Kanagawa Cancer Center Biospecimen Center. Case 1 involved a 45-year-old woman with two masses, measuring 3.5 cm in the upper central area of the right breast and 1.0 cm in the axilla. Histologic diagnosis of the breast mass was mastitis based on core-needle biopsy (CNB) and incisional biopsy. The lesions decreased with steroid treatment; however, they grew rapidly 14 months after the CNB. The patient was diagnosed with metastatic carcinoma based on the resected axillary mass and underwent Halsted mastectomy and axillary lymph node dissection. Beneath the nipple, an ill-defined breast mass measuring 6.0 × 5.5 × 4.5 cm invading the dermis, nipple, and greater pectoral muscle was detected; an axillary mass measuring 5.0 × 3.5 × 4.5 cm and multiple lymph node metastases were also observed. The final histopathologic diagnosis was mixed metaplastic carcinoma (60% low-grade adenosquamous carcinoma and 40% squamous cell carcinoma). One month after surgery, skin, liver, and lymph node metastases appeared. Chemotherapy failed, and the patient died due to cancer at 9 months after surgery. The clinicopathologic characteristics of Cases 2, 3, and 4 are summarized in Table S1.

The study was conducted according to the Declaration of Helsinki and was approved by the Ethics Committee of the Kanagawa Cancer Center (Approval No. H28-240). Written informed consent for retrospective studies, including somatic and germline genetic analyses, as broad comprehensive consent, was obtained from the patients.

### Histopathologic and immunohistochemical analyses

The histology and histologic grade of tumors were evaluated based on the fifth edition of World Health Organization Classification of Tumors [1]. Immunohistochemistry was performed on sections from a representative paraffin block of each tumor. The detailed methods are provided in the Supplementary Information. Expression of estrogen receptor (ER), progesterone receptor (PgR), human epidermal growth factor receptor 2 (HER2), p40, SMA, Ki-67, and SMAD4 was investigated using immunohistochemistry, and expression levels of ER, PgR, and HER2 were evaluated based on the latest American Society of Clinical Oncology/College of American Pathologists guidelines [17, 18].

### Whole-genome sequencing and subsequent standard analyses

DNA was extracted from surgically resected specimens and stored in our biobank. For Case 1, primary breast tumor tissue specimens consisting of LGASC and axillary lymph node metastasis specimens consisting of MSC (LNMSC) were processed separately. For Cases 2, 3, and 4, breast tumor tissues consisting of MSC, referred to as M2T, M3T, and M4T, respectively, were evaluated. Tumor content was confirmed by hematoxylin and eosin (HE) staining of frozen sections. Reference genomic DNA was extracted from healthy skin samples from each patient. Primary data analysis of whole-genome sequencing (WGS) was performed by Genewiz (Tokyo, Japan). Subsequent analyses for single-nucleotide variants (SNVs), short insertions and deletions, and structural variations were analyzed using the Genomon 2 DNA analysis pipeline (https://github.com/Genomon-Project) at Human Genome Center, the Institute of Medical Science, University of Tokyo (Tokyo, Japan). Copy number variations were analyzed using DNAcopy version 1.56.0. (https://bioconductor.org/packages/release/bioc/html/DNAcopy.html), and GISTIC 2.0 [19]. The detailed methods are provided in the Supplementary Information.

### Analysis of clonal evolution of cancer in Case 1

MesKit version 1.6.0, an R package [20], was used to analyze clonal evolution of LGASC and LNMSC based on WGS datasets.

Sclust [21] was used to estimate copy number, purity, and cancer cell fraction (CCF) based on these values. The detailed methods are provided in the Supplementary Information.

### Reverse transcription polymerase chain reaction

Presence of the *SMAD4::DCC* fusion transcript in LGASC and LNMSC was evaluated using reverse transcription (RT)-polymerase chain reaction (PCR). Briefly, total RNA extracted from frozen tissue samples of LGASC and LNMSC was reverse transcribed using SuperScript IV VILO Master Mix (Invitrogen, Waltham, MA) and amplified by PCR using PrimeSTAR HS DNA polymerase (Takara, Kyoto, Japan) and the primers listed in the Supplementary Information. The amplified fragments were subcloned into vectors and sequenced using an ABI PRISM 3130xl genetic analyzer (Thermo Fisher Scientific).

### Western blotting

The presence of the *SMAD4::DCC* chimeric protein was determined using Western blotting with rabbit and mouse monoclonal antibodies that recognize the N-terminal region of SMAD4 and the C-terminal region of DCC, respectively. Anti-vinculin was used as a loading control. Signal detection was performed using the ImmunoStar LD-enhanced chemiluminescence detection reagent (Fujifilm Wako Chemicals, Osaka, Japan). The detailed methods are provided in the Supplementary Information.

### Statistical analysis

Genetic data were compared using the Mann–Whitney *U* test. *P* values of less than 0.05 were used to denote statistical significance. All statistical analyses were performed using R version 4.0.2.

## Results

### Pathological findings

**Fig. 1:**
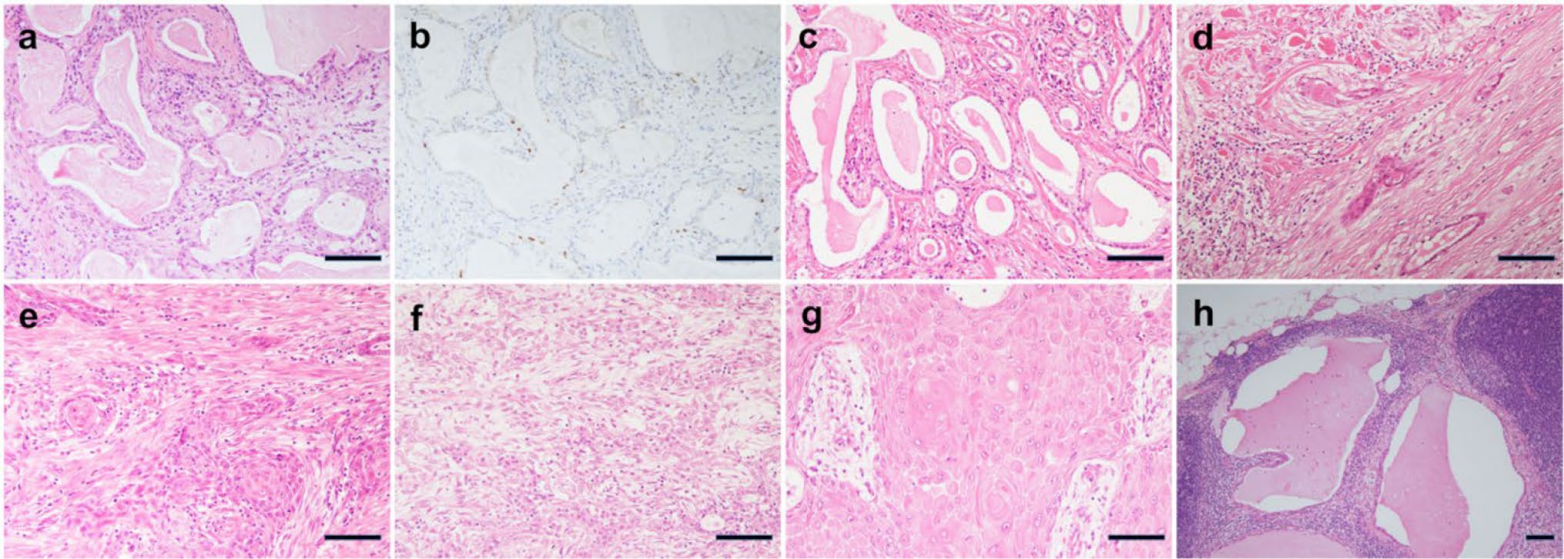
Histological and immunohistochemical features of LGASC with high-grade transformation (Case 1). **a** and** b** Histological features of core-needle biopsy. **a** The slide shows irregular dilation of the ducts with secretion in the lumen and moderate chronic inflammation in the stroma. **b** The immunohistochemical study showed that p40-positive cells existed discontinuously in the periphery of the ducts. **c**–**h** Histological findings of mastectomy specimens. **c** and **d** The superficial area. **c** The superficial area of the tumor consists of infiltrative glandular structures filled with abundant secretory materials in the lumen, resembling secretory carcinoma. The nuclear atypia of the tumor cells is bland. Few cells have mucin in their cytoplasm. **d** A view of a few tumor nests reveals squamous differentiation. **e** The deep area. The tumor displays clearer squamous cell differentiation. Some gland formations in the nests are seen. These findings are compatible with squamous cell carcinoma with the adenosquamous carcinoma pattern. **f** and **g** Histological findings of an axillary mass. **f** The lesions consisted of adenosquamous and spindle cell carcinoma. **g** Pure squamous cell carcinoma with a keratinization component is seen. **h** Some metastatic foci in the dissected lymph nodes include the LGASC component. Scale bar = 0.1 mm. LGASC, low-grade adenosquamous carcinoma

In Case 1, the initial CNB and incisional biopsy specimens showed irregular duct dilation with bland-appearing cuboidal cells and luminal secretion. Mitoses were uncommon. Immunohistochemistry identified p40-positive cells existing discontinuously in the periphery of the ducts (Fig. 1a, b). The background showed moderate inflammation in the stroma. In the later mastectomy specimen, histology of the superficial area of the breast tumor was similar to that observed in preceding biopsies (Fig. 1c). In addition, a few tumor nests displayed squamous differentiation (Fig. 1d). Mucin-containing cells were limited. Some bland spindle cells surrounding the tumor were p40 negative and weakly SMA positive. These findings are consistent with LGASC. In the deep area, the tumor displayed transition to MSC with a high-grade squamous cell carcinoma component with spindle cell morphology (Fig. 1e). LGASC and high-grade carcinoma were mixed with each component at the border, and the boundary was indistinct. The breast tumor contained slightly more LGASC components than high-grade components. The axillary mass was an extranodal invasion of the high-grade component of the breast tumor with a greater number of spindle cells and a pure keratinizing squamous cell carcinoma component than the breast tumor (Fig. 1f, g). Some metastatic foci in the dissected lymph nodes other than the axillary mass also included an LGASC component (Fig. 1h). ER and PgR were negative, and the HER2 score was 1 + in all tumor areas.

### Somatic mutations and copy number variants

**Fig. 2:**
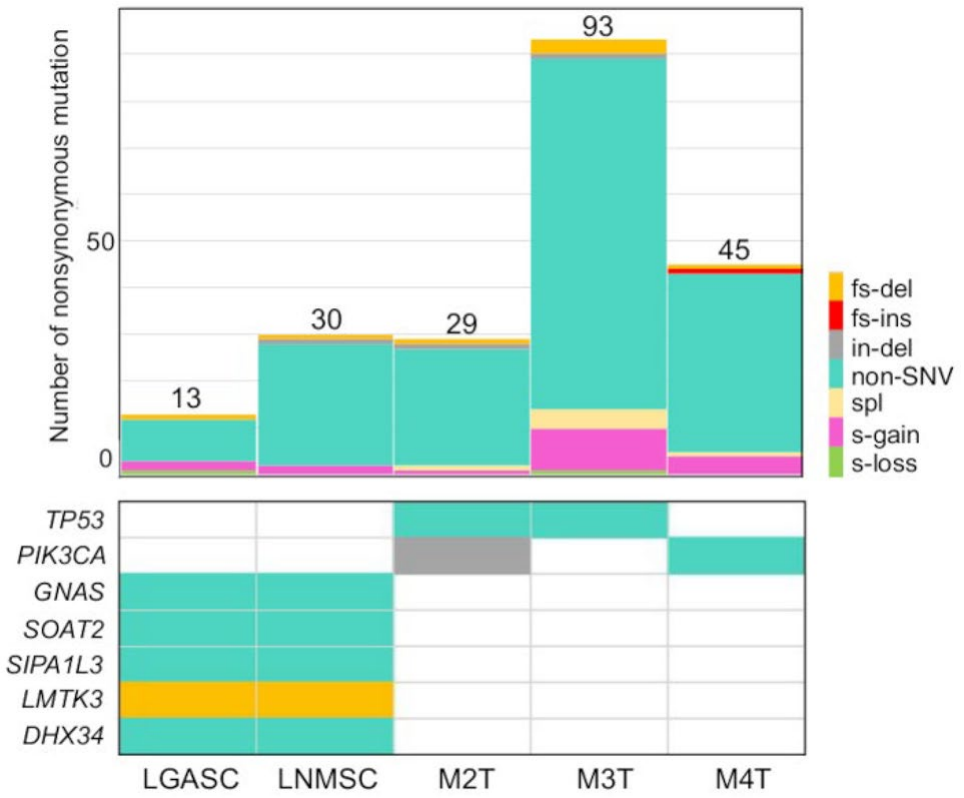
Summary of nonsynonymous somatic mutations (SNVs, short indels, and splice site mutations) and recurrently appearing mutations in five metaplastic carcinoma samples. The upper bar chart demonstrates the numbers and predicted functional consequences of the nonsynonymous somatic mutations per metaplastic carcinoma sample. The lower table demonstrates the mutations that recurrently appeared among the samples. The symbols of the mutated genes are shown on the left side, and the names of the samples are provided at the bottom of each column. SNV, single-nucleotide variant; fs-del, frameshift deletion; fs-ins, frameshift insertion; in-del, in-frame deletion; non-SNV, nonsynonymous single-nucleotide variant; spl, splicing mutation; s-gain, stop-gain mutation; s-loss, stop-loss mutation; LGASC, low-grade adenosquamous carcinoma; LNMSC, lymph node metastasis consisting of high-grade metaplastic carcinoma of the breast with a predominant metaplastic squamous cell carcinoma component

The average sequencing coverage was 33.8 for the five tumor samples and 34.3 for the healthy skin samples. The tumor purity of LGASC, LNMSC, M2T, M3T, and M4T estimated by Sclust [21] was 0.20, 0.20, 0.42, 0.39, and 0.24, respectively. All somatic mutations satisfying the criterion set are listed in Tables S2–S6. Pathogenic mutations, which are also driver gene mutations in breast cancer, are summarized in Table 1. Recurrent nonsynonymous somatic mutations are shown in Fig. 2. Five mutations were shared between the LGASC and LNMSC cases, and only the *GNAS* mutation c.C2530T:p.Arg844Cys, a known mutational hotspot for this gene, was considered a pathogenic variant [22]. Furthermore, only de novo MSC harbored pathogenic *TP53* or *PIK3CA* mutation.

**Table 1:** Summary of somatic mutations (SNVs, short indels) in the five metaplastic carcinomas analyzed in this study

Sample	Number of all mutations	Number of nonsynonymous mutations	Pathogenic nonsynonymous mutations
LGASC	275	13	*GNAS*(c.C2530T:p.R844C)^a^
LNMSC	1118	30	*GNAS*(c.C2530T:p.R844C)^a^
M2T	2233	29	*TP53*(c.C535T:p.H179Y)^a^
			*PIK3CA*(c.1255-1260del:p.419-420del)
M3T	5167	93	*TP53*(c.C318G:p.S106R)
M4T	2609	45	*PIK3CA*(c.G1624A:c.p.E542K)^a^
			*ERBB2*(c.G2329T:p.V777L)^a^
			*HRAS*(c.G34A:p.G12S)^a^

LGASC, low-grade adenosquamous carcinoma; LNMSC, lymph node metastasis consisting of high-grade metaplastic carcinoma of the breast with a predominant metaplastic squamous cell carcinoma component

^a^Hotspot mutations

**Fig. 3:**
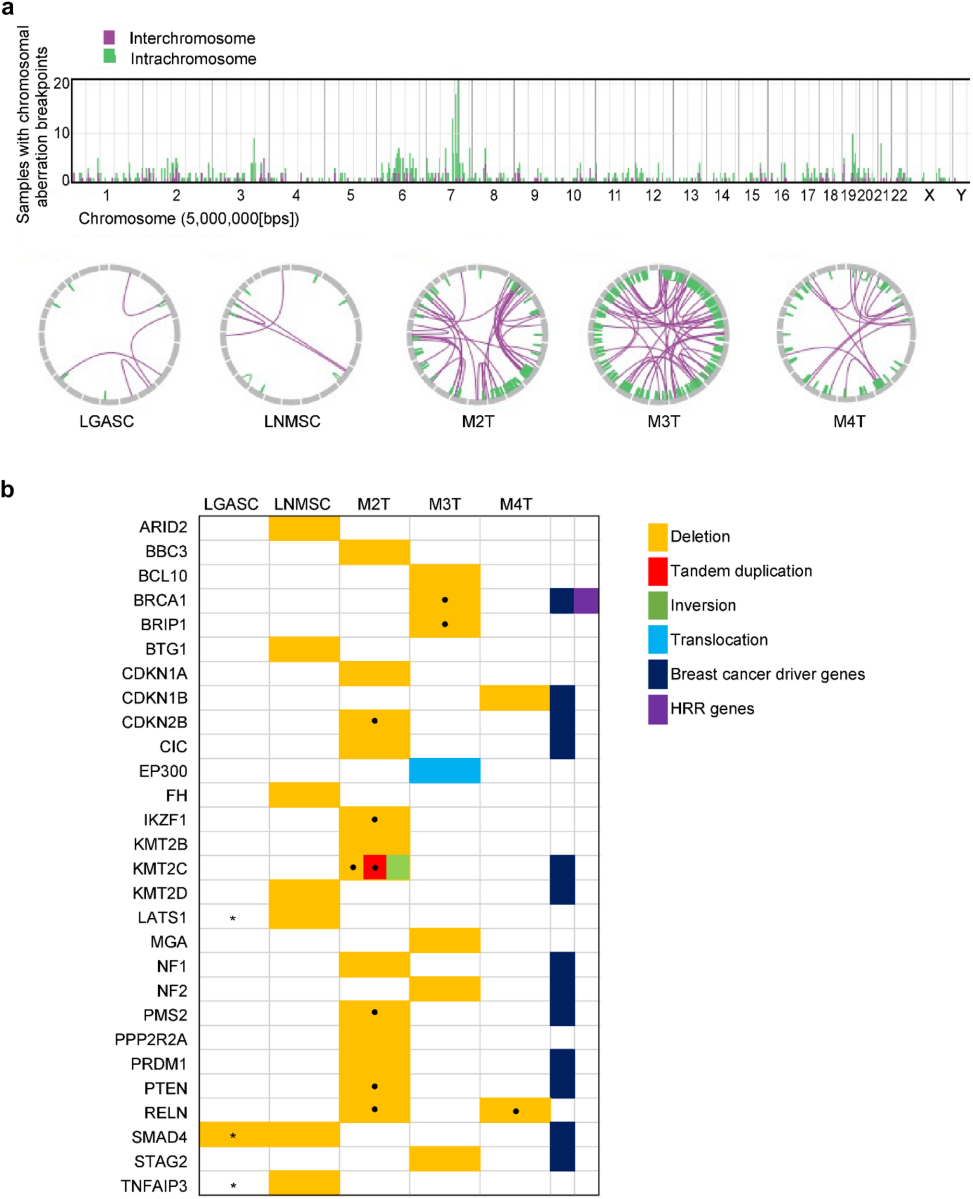
Summary of the SVs of the five metaplastic carcinomas.** a** The bar graph in the upper part represents the number of breakpoints of SVs per chromosome for the five samples. The Circos plots in the lower part show two breakpoints connected by a line for each metaplastic carcinoma. **b** The genes affected by SVs (deletion or tandem duplication or fusion) were curated as oncogenic or likely oncogenic in the OncoKB database. The type of SV is color-coded as stated in the legend. The dot in the box indicates that multiple SVs are identified in the corresponding genes. Asterisks in the boxes of LGASC indicate that LGASC has few reads representing the same deletion as LNMSC confirmed using Integrative Genomics Viewer, which is not recognized by the Genomon 2 DNA analysis pipeline because of the threshold. Namely, LGASC not only showed *SMAD4* deletion in intron 1 but also a read indicating a *SMAD4::DCC* fusion gene identical to that found in LNMSC. SV, structural variation; LGASC, low-grade adenosquamous carcinoma; LNMSC, lymph node metastasis consisting of high-grade metaplastic carcinoma of the breast with a predominant metaplastic squamous cell carcinoma component; HRR, homologous recombinational repair

**Fig. 4:**
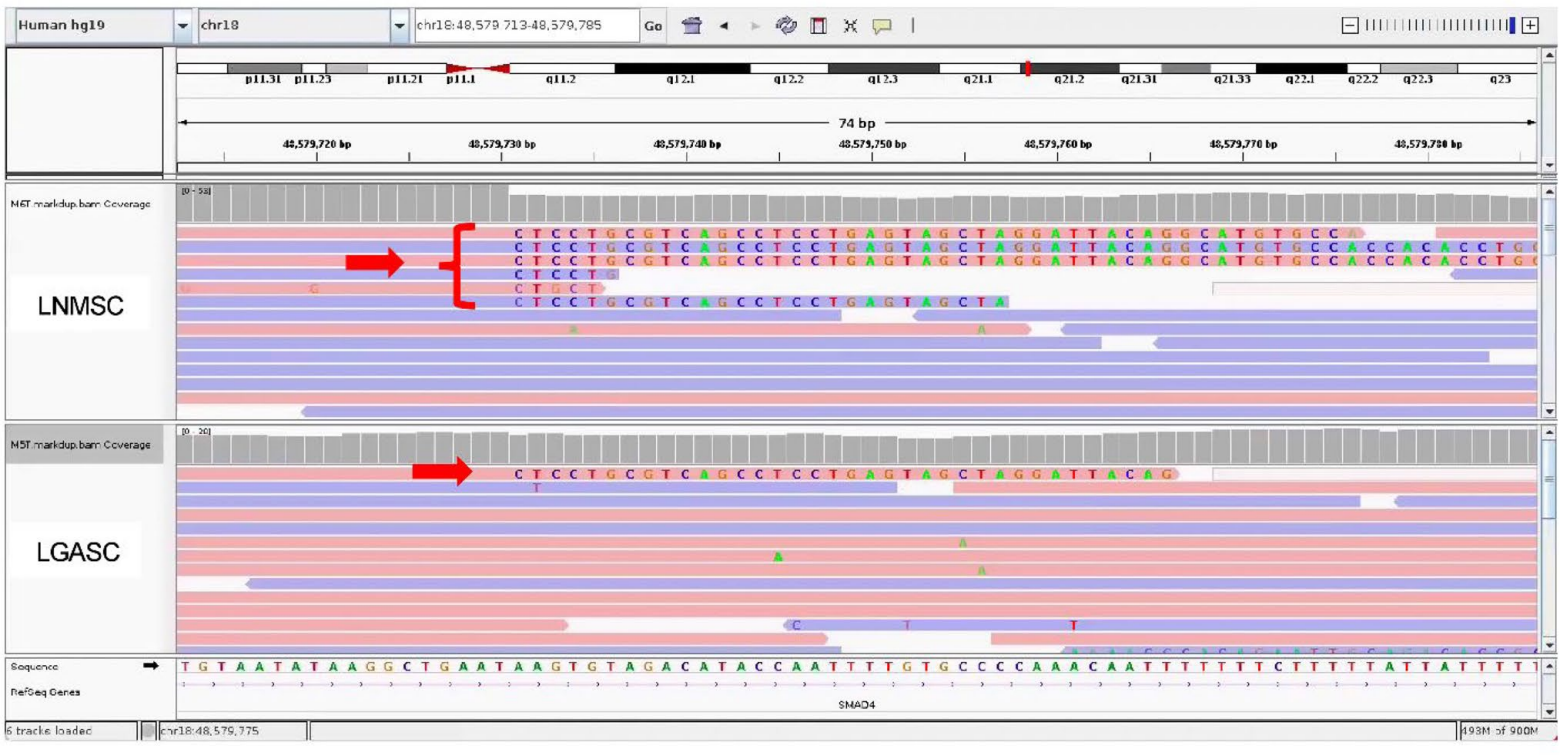
Integrative Genomics Viewer data show the reads corresponding to *SMAD4::DCC* fusion in LGASC and LNMSC. In intron 4 of the *SMAD4* gene (position on a chromosome is 48,579,729), the red arrow points to the reads having soft-clipping bases. Supplementary alignments of these reads start from position 50,300,003, corresponding to *DCC* intron 3. The upper part represents LNMSC and the lower part LGASC. Only one corresponding read was identified in LGASC. LGASC, low-grade adenosquamous carcinoma; LNMSC, lymph node metastasis consisting of high-grade metaplastic carcinoma of the breast with a predominant metaplastic squamous cell carcinoma component

All SVs identified in the present study are shown in Tables S7–S11 and schematically summarized in Fig. 3a. The number of SVs in the LGASC and LNMSC was smaller than that in the three de novo MSCs (9 and 12 vs. 124, 250, and 107, respectively); however, the difference was not statistically significant (*P* = 0.2 by the Mann–Whitney *U* test). We defined the genes located at the breakpoints of SVs or between the breakpoints of deletions as genes affected by SVs. Subsequently, we listed the genes with SVs (deletion, tandem duplication, or fusion) that are curated as oncogenic or likely oncogenic in the OncoKB database (Memorial Sloan Kettering Cancer Center; https://www.oncokb.org/) (Fig. 3b). Compared with LGASC, the LNMSC contained deletions in multiple tumor-suppressor genes, such as *KMT2D* and *BTG1*. At chromosome 18q (chr18q), the LGASC harbored a 33 bp deletion in intron 1 of *SMAD4*, whereas the LNMSC harbored a large deletion spanning from chr18:48,579,729 in intron 4 of *SMAD4* to chr18:50,300,003 in intron 2 of *DCC* (hg19), which may generate a *SMAD4::DCC* fusion gene. When confirming the genomic data using Integrative Genome Viewer, only a few reads corresponding to the same *SMAD4::DCC* fusion (Fig. 4) or to deletion of *TNFAIP3* or *LATS1* were also found in the LGASC. No genetic events satisfying the criteria of chromothripsis were found in any of the specimens.

Copy number analysis of the five MBCs using DNAcopy showed GISTIC peaks of copy number gain at 8q24.3 (M2T and M3T) (Fig. S1), but no significant GISTIC peaks of copy number loss were identified. On the other hand, Sclust predicted copy number loss with loss of heterozygosity (LOH) at 9p and 18q in the LGASC and LNMSC; the identified 18q segment includes *SMAD4* and a part of *DCC* (Fig. S2).

### Analysis of SMAD4::DCC fusion and SMAD4 expression in low-grade adenosquamous carcinoma and its associated high-grade metaplastic carcinoma

**Fig. 5:**
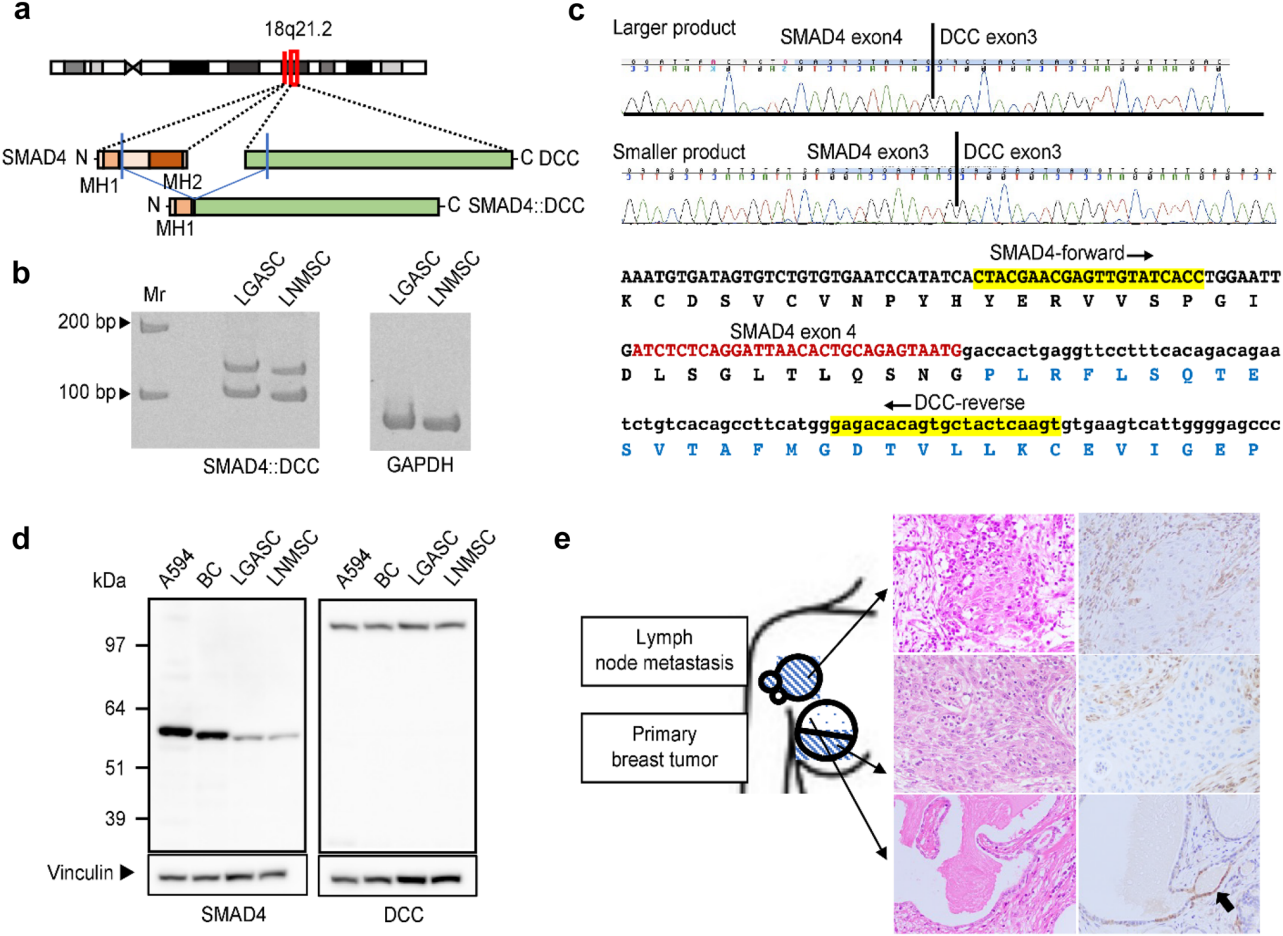
*SMAD4::DCC* fusion gene in LGASC and LNMSC. **a** The predicted SMAD4::DCC chimeric protein is schematically indicated. It is considered that the MH1 domain of SMAD4 is retained but that the MH2 domain of SMAD4 is lost. **b** RT‒PCR demonstrated the presence of two *SMAD4::DCC* fusion transcripts of different sizes in LGASC and LNMSC. On the right, two lanes show the internal glyceraldehyde 3-phosphate dehydrogenase control. Molecular weight markers are indicated at the left. The forward PCR primer targets exon 3 of *SMAD4* (*SMAD4*-forward) and the reverse primer exon 3 of *DCC* (*DCC*-reverse). **c** Sanger sequencing revealed two chimeric transcripts of different sizes in both LGASC and LNMSC; the larger product is the *SMAD4* exon 4–*DCC* exon 3 fusion transcript, and the smaller product is the *SMAD4* exon 3–*DCC* exon 3 fusion transcript. **d** Western blotting with antibodies recognizing the N-terminus of SMAD4 (left) and the C-terminus of DCC (right) confirmed the existence of apparently wild-type SMAD4 and apparently wild-type DCC but not a protein derived from the *SMAD4::DCC* fusion. Molecular weight markers are indicated at the left. Vinculin as the loading control is shown at the bottom. On the left, two lanes show the positive controls (A594, lung cancer cell line; BC, breast cancer tissue). **e** Immunohistochemical study using an anti-SMAD4 C-terminus antibody. Schematic diagram (left) of the primary tumors and axillary mass arising from lymph node metastases shows locations where surgically resected tissues were analyzed using immunohistochemistry. The blue dot and diagonal stripe patterns represent the histology of LGASC and high-grade metaplastic carcinoma with a predominant squamous cell carcinoma component, respectively. LNMSC and LGASC samples were collected from metastatic lesions in the lymph node and superficial area of the mammary tumor, respectively. Immunohistochemical staining for SMAD4 (right) corresponding to hematoxylin and eosin-stained images (middle) shows weak positivity for SMAD4 in the nuclei of a few glandular epithelial cells in the LGASC (black arrow in bottom row). SMAD4 was mostly negative in high-grade metaplastic carcinoma with a predominant squamous cell carcinoma component (upper and middle rows). Nuclei of background stromal cells were positive. LGASC, low-grade adenosquamous carcinoma; LNMSC, lymph node metastasis consisting of high-grade metaplastic carcinoma of the breast with a predominant metaplastic squamous cell carcinoma component; RT‒PCR, reverse transcription polymerase chain reaction

WGS analysis revealed the possibility that the *SMAD4::DCC* fusion transcript and protein were produced in the LNMSC (Fig. 5a). RT‒PCR revealed two clear fragments (Fig. 5b), and the nucleotide sequence indicated that both derive from *SMAD4::DCC* fusion transcripts. The larger product was found to contain exons 3 and 4 of *SMAD4* and exon 3 of *DCC*, whereas the smaller product did not include exon 4 of *SMAD4* (Fig. 5c). Nonetheless, the predicted SMAD4::DCC fusion protein was not detected by Western blotting (Fig. 5d). The immunohistochemical study using an anti-SMAD4 antibody that recognizes the C-terminus of SMAD4 showed weak positivity in a few nuclei of the glandular epithelium in LGASC, but it was mostly negative in the MSC associated with LGASC (Fig. 5e).

### Clonal evolution from low-grade adenosquamous carcinoma to high-grade metaplastic carcinoma

**Fig. 6:**
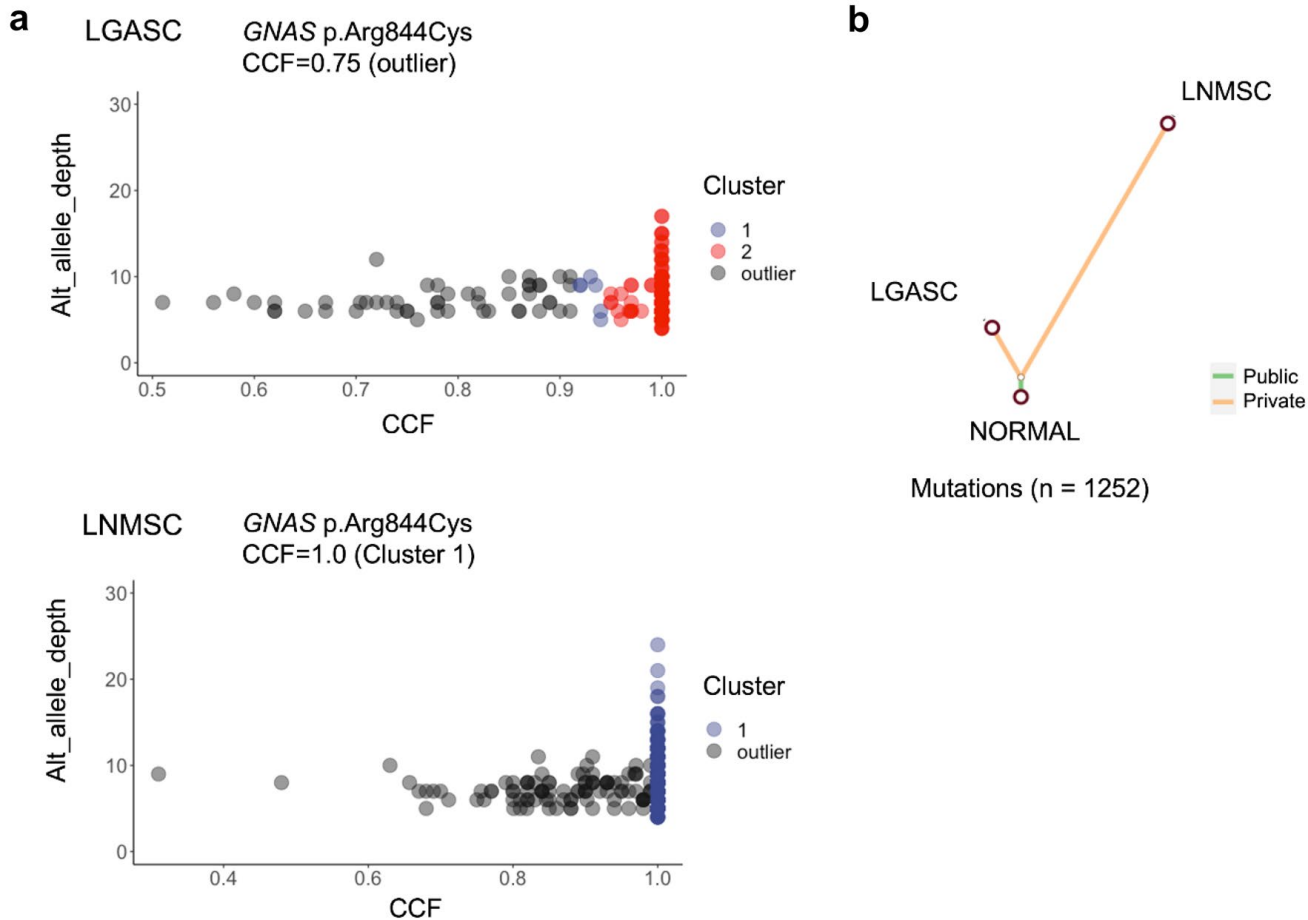
Clonal evolution analysis of LGASC and LNMSC using MesKit. **a** Mutation clustering of LGASC and LNMSC, which developed in the same patient, was performed based on a Gaussian finite mixture model using MesKit. Each dot corresponds to a single mutation, and the horizontal dimension shows the cancer cell fraction (CCF). The vertical dimension was modified and expanded from the original output, showing the depth of mutations. **b** A phylogenetic tree of the two tumor components was constructed from somatic SNVs and indels using the neighbor-joining algorithm. Branches are colored according to the regional distribution of mutations. “Public” mutations exist in all regions of the tumor, whereas “Private” mutations exist in a single region. The branch lengths are proportional to the number of mutations. LGASC, low-grade adenosquamous carcinoma; LNMSC, lymph node metastasis consisting of high-grade metaplastic carcinoma of the breast with a predominant metaplastic squamous cell carcinoma component; CCF, cancer cell fraction; SNV, single-nucleotide variant

The SNVs and indels of Case 1 were clustered by the CCFs using MesKit based on the Gaussian finite mixture model. The results yielded two clusters for LGASC and one for LNMSC (Fig. 6a). The CCF was more widely distributed in LGASC than in LNMSC. The estimated CCF of the *GNAS* mutation (c.C2530T:p.Arg844Cys) was 0.75 in the LGASC, which was an outlier in the clustering, indicating that few cells harbored this mutation. Conversely, the estimated CCF of the *GNAS* mutation was 1.0 in the, forming a significant cluster for this tumor. Phylogenic tree analysis revealed clear divergence of LGASC and LNMSC (Fig. 6b). In general, LNMSC accumulated more mutations after branching than LGASC.

## Discussion

In this study, we demonstrated the genetic profile of an LGASC that differentiated to high-grade MBC with clonal concentration and accumulation of loss-of-function mutations in multiple tumor-suppressor genes. The converted high-grade MBC was genetically different from de novo high-grade MBC.

Progression from LGASC to high-grade MBC is a rare phenomenon, with 10 cases reported. Among cases with known histology of high-grade components, spindle cell carcinoma was found to be the most common, with squamous cell carcinoma in two cases [8–12]. Prognostic information was available for 3 of 10 cases, and metastasis or cancer death was not reported during follow-up periods ranging from less than 6 months to 11 years [8, 12]. One molecular study about transformation has been conducted. Geyer et al. [10] examined the CNV of one LGASC case transitioning to high-grade MBC consisting of malignant spindle cells. This LGASC case involved a rather simple molecular karyotype without amplification based on microarray comparative genomic hybridization. In contrast, an LGASC case with progression to high-grade MBC displayed a large degree of genomic complexity, with high-level amplification of 7p11.2, encompassing *EGFR*, and 7q11.21. Chromogenic in situ hybridization for *EGFR* showed spindle cells harboring *EGFR* amplification in both the low- and high-grade areas of this tumor.

In our study, multiple SNVs, SVs, and CNVs common to both LGASC and LNMSC proved that LGASC and its associated MSC have common clonal ancestry; cancer clonal evolution analysis findings also support this concept. The results of clustering by CCF suggested a decrease in intratumoral heterogeneity and an increase in the fraction of tumor cells harboring the *GNAS* mutation, indicating clonality during progression from LGASC to MSC. Moreover, LNMSC displayed additional deletions of tumor-suppressor genes, such as *KMT2D* and *BTG1*. KMT2D functions as an epigenetic modulator that methylates lysine residue 4 on the tail of histone H3 (H3K4) [23]. *BTG1* is a member of an antiproliferative gene family, and the protein encoded regulates cell growth and differentiation [24]. Some studies have indicated that low *KMT2D* transcript levels or decreased BTG1 protein expression are associated with poor survival in breast cancer [25, 26]. These results support the notion that clonal selection, with enrichment of tumor cells with *GNAS* gene alteration, and stepwise inactivation of tumor-suppressor genes rather than addition of other driver gene activation promotes progression to high-grade MBC from LGASC. High-grade MBC derived from LGASC and de novo high-grade MBC have similar squamous morphology; however, the former has fewer SVs and does not harbor *TP53* loss-of-function mutations. This is consistent with the results of previous studies [4, 8, 13, 14, 16]. There are several speculations regarding the cellular origin of metaplastic carcinoma, such as dedifferentiation from conventional adenocarcinoma or development from basal-like stem cells. Metaplastic carcinoma with few SVs and lack of *TP53* mutations might derive from the low-grade variant of MBC, as in the present case.

One of the most frequent regions with LOH in breast cancer is 18q, and *SMAD4* and *DCC* located at 18q21 are inactivating tumor-suppressor gene candidates [27]. According to PanCancer Atlas, a few homozygous codeletions of *DCC* and *SMAD4* have been reported in breast cancer (https://www.cbioportal.org). We found a novel *SMAD4::DCC* fusion gene generated by deletion in an LGASC and its associated MSC using WGS. Although *SMAD4::DCC* fusion transcripts were detected by RT‒PCR, no fusion protein was identified by Western blotting. Possible causes of these results are that the fusion transcript might not be efficiently translated due to currently unknown mechanisms or that the fusion protein might be easy to degrade if produced. The Sclust results suggest the possibility of LOH at 18q in both LGASC and LNMSC, which is consistent with the absence of SMAD4 protein expression in LNMSC by immunohistochemistry. Conversely, although few reads exhibiting the *SMAD4::DCC* deletion were found for LGASC, which was also observed for LNMSC, the cause of low SMAD4 protein expression was unclear. Possible causes of this reduced expression are epigenetic modification and protein degradation. The already reduced SMAD4 protein expression in LGASC may be involved in the tumorigenesis of LGASC.

LGASC is generally considered as an indolent tumor, while two cases of metastasis, one to lymph node and another to lung, and 5 locally recurrent cases after excisional biopsy were reported thus far [6]. One locally recurrent case was a fatal disease with invasion to the thorax, but no studies investigating the genetic background of progression have been yet conducted. The present study describes a patient having LGASC and metastatic high-grade MSC sharing a common *GNAS* pathogenic mutation and *SMAD4::DCC* fusion; the findings raise the possibility that clonal selection for the concentration of driver gene mutation and tumor-suppressor gene inactivation may participate in the mechanism of progression. Given the rarity of LGASC and its progression to high-grade MBC, we were able to examine only one such case precisely; whether the result can be generalized to the genetic mechanism of malignant progression from LGASC to high-grade MBC remains uncertain. Furthermore, because the present case involved a *GNAS* mutation instead of a *PIK3CA* pathogenic mutation, which is frequent in LGASC [4, 8, 16], this case might be exceptional. There must be differences in mechanisms for LGASC progression, and further investigations with as many additional cases as possible are needed to clarify this issue.

### Supplementary Information

Below is the link to the electronic supplementary material.

Supplementary file 1 (DOCX 52 KB)

Supplementary file 2 (PDF 898 KB)

Supplementary file 3 (XLS 32 KB)

Supplementary file 4 (XLS 2196 KB)

Supplementary file 5 (XLS 138 KB)

## Data Availability

All data generated and analyzed during this study are included in this published article and its supplementary information files.

## Code availability

The datasets generated during and/or analyzed during the current study are available from the corresponding author on reasonable request.
