# Supplementary material for: Genetic analysis of low-grade adenosquamous carcinoma of the breast progressing to high-grade metaplastic carcinoma
Source: Breast Cancer Res Treat. 2023 Aug 31;202(3):563–73. doi: 10.1007/s10549-023-07078-9 (PMC10564816; doi:10.1007/s10549-023-07078-9)
Supplement: Supplementary file 1 — (DOCX 52 KB) [file 10549_2023_7078_MOESM1_ESM.docx]

**Breast cancer research and treatment**

**Genetic analysis of low-grade adenosquamous carcinoma of the breast progressing to high-grade metaplastic carcinoma**

Kae Kawachi^*,1,2^, Xiaoyan Tang^*,3^, Rika Kasajima^*,4,5^, Takashi Yamanaka^6^, Eigo Shimizu^5^, Kotoe Katayama^5^, Rui Yamaguchi^5,7,8^, Kazuaki Yokoyama^9^, Kiyoshi Yamaguchi^10^, Yoichi Furukawa^10^, Satoru Miyano^5,11^, Seiya Imoto^5^, Emi Yoshioka^1^, Kota Washimi^1^, Yoichiro Okubo^1^, Shinya Sato^4^, Tomoyuki Yokose^1^, Yohei Miyagi^4^

^1^Department of Pathology, Kanagawa Cancer Center, 2-3-2 Nakao, Aasahi-ku, Yokohama, Japan

^2^Department of Pathology, The Jikei University School of Medicine, 3-25-8 Nishishinbashi, Minato-ku, Tokyo, Japan

^3^Department of Pathology, Nihon University Hospital, 1-6 Kandasurugadai, Chiyoda-ku, Tokyo, Japan

^4^Molecular Pathology and Genetics Division, Kanagawa Cancer Center Research Institute, 2-3-2 Nakao, Aasahi-ku, Yokohama, Japan

^5^Division of Health Medical Intelligence, Human Genome Center, Institute of Medical Science, The University of Tokyo, 4-6-1 Shirokanedai, Minato-ku, Tokyo, Japan

^6^Department of Breast and Endocrine Surgery, Kanagawa Cancer Center, 2-3-2 Nakao, Aasahi-ku, Yokohama, Japan

^7^Division of Cancer Systems Biology, Aichi Cancer Center Research Institute, 1-1 Kanokoden, Chikusa-ku, Nagoya, Japan

^8^Division of Cancer Informatics, Nagoya University Graduate School of Medicine, 65 Tsurumai-cho, Showa-ku, Nagoya, Japan

^9^Department of Hematology/Oncology, Research Hospital, Institute of Medical Science, The University of Tokyo, Tokyo, Japan

^10^Division of Clinical Genome Research, Institute of Medical Science, The University of Tokyo, 4-6-1 Shirokanedai, Minato-ku, Tokyo, Japan

^11^Department of Integrated Data Science, Medical and Dental Data Science Center, Tokyo Medical and Dental University, 2-3-10 Kandasurugadai, Chiyoda-ku, Tokyo, Japan

**Corresponding author**

Yohei Miyagi

E-mail address: miyagi@gancen.asahi.yokohama.jp

**Supplementary information**

**1. Detailed materials and methods**

**2. Supplementary figure and table legends**

**1. Detailed materials and methods**

***Histopathologic and immunohistochemical analyses***

Surgically resected tissues were fixed in 10% buffered formalin, embedded in paraffin, and stained with hematoxylin and eosin. The histology and histological grade of the tumors were evaluated based on the fifth edition of World Health Organization Classification of Tumours [1]. Immunohistochemical staining was performed on a section from a representative paraffin block of each tumor. Then, heat-induced antigen retrieval was performed. Primary antibodies against the following were used: estrogen receptor (ER) (SP1, Roche, Basel, Switzerland, prediluted), progesterone receptor (PgR) (1E2, Roche, prediluted), human epidermal growth factor receptor 2 (HER2) (4B5, Roche, prediluted), p63 (4A4, Nichirei, Tokyo, Japan, prediluted), p40 (BC20, Nichirei, prediluted), SMA (1A4, Roche, prediluted), Ki-67 (MIB-1, Agilent, Santa Clara, CA, USA, prediluted), and SMAD4 (EP618Y, Abcam, Cambridge, UK, 1:100). All immunohistochemical procedures, except for p40 and SMAD4, were performed using the automatic staining machine VENTANA BenchMark ULTRA IHC/ISH system (Roche). ER, PgR, and HER2 were detected using iVIEW DAB Detection Kit (Roche); p63, SMA, and Ki-67 were detected using ultraView Universal DAB Detection Kit (Roche). For p40, immunostaining was performed using HISTOSTAINER 48A (Nichirei), and its expression was detected using the Histofine Simple Stain MAX-PO kit (Nichirei). For SMAD4, immunostaining was performed using BOND-III (Leica Biosystems, Nussloch, Germany). Positive and negative controls were used. ER, PgR, and HER2 were evaluated according to the current American Society of Clinical Oncology/College of American Pathologists guidelines [2, 3].

***Whole-genome sequencing***

Frozen tissue samples from surgically resected specimens were used. In Case 1, primary breast tumor tissue consisting of low-grade adenosquamous carcinoma (LGASC) and axillary lymph node metastasis consisting of high-grade metaplastic carcinoma of the breast with a predominant metaplastic squamous cell carcinoma component (LNMSC) were analyzed separately. In Cases 2, 3, and 4, tumor tissues consisting of high-grade metaplastic carcinoma of the breast with a predominant metaplastic squamous cell carcinoma component (MSC) were collected (M2T, M3T, and M4T, respectively). Hematoxylin and eosin-stained sections of these frozen samples confirmed tumor histology. Furthermore, matching healthy skin tissues were collected as a reference. Genomic DNA was extracted from frozen MBC tissue and matched with healthy skin tissues using a standard method with protease K (PK) digestion. Briefly, frozen tissues were minced and incubated at 65°C overnight in 1 mg/mL PK solution containing 10 mM Tris-HCl buffer (pH 7.8–8.0) (Thermo-Fisher Scientific, Inc., Waltham, MA), 0.4% (w/v) sodium dodecyl sulfate (Nippon Gene Co., Ltd., Tokyo, Japan), 150 mM NaCl, and 10 mM ethylenediaminetetraacetic acid (Thermo-Fisher Scientific, Inc.). The solution was extracted in 25:24:1 phenol/chloroform/isoamyl alcohol (phenol-CIAA) solution (FUJIFILM Wako Chemicals) several times; the DNA was precipitated from the aqueous phase with ethanol and collected by centrifugation. The DNA was dissolved in autoclaved distilled water (dH_2_O) and treated with 10 µg/mL DNase-free RNase (Pure Link RNase A, Thermo-Fisher Scientific) at 37°C for 30 min. The sample was treated once with phenol-CIAA, and the DNA was obtained as described above. The double-stranded DNA was quantified with a Qubit 2 (Thermo-Fisher Scientific), and DNA purity was evaluated using a NanoPhotometer (Implen, Munich, Germany) as A260/A280 and A260/230 optical density ratios. Whole-genome sequencing (WGS) was carried out by Genewiz (Shinagawa-ku, Tokyo, Japan) for primary data analysis. Sequencing libraries were constructed from 100 ng of extracted DNA using the TruSeq Nano DNA Library Prep kit (Illumina, Hayward, CA) and sequenced using a standard 150-bp paired-end read protocol. The obtained sequence data were analyzed using the Genomon 2 DNA analysis pipeline (https://github.com/Genomon-Project) at Human Genome Center, Institute of Medical Science, University of Tokyo (Tokyo, Japan).

***Analysis of single-nucleotide variants and short insertions and deletions (indels)***

Single-nucleotide variants (SNVs) with minimum depth ≥8, base quality ≥15, variant read ≥4, P <0.01 (Fisher’s exact test), and variant allele frequency ≥0.02 in the tumor were first selected for analysis. SNVs in protein-coding regions that result in changes in amino acid sequences were further evaluated to identify pathogenic or likely pathogenic ones by interrogating against Catalog of Somatic Mutations in Cancer (COSMIC) (https://cancer.sanger.ac.uk/cosmic), 1000 Genomes Project (https://international genome.org), dbSNPs (NCBI, NIH; https://www.ncbi.nlm.nih.gov/snp/), Functional Analysis through Hidden Markov Models (v2.3) (http://fathmm.biocompute.org.uk), ClinVar (NCBI, NIH; https://www.ncbi.nlm.nih.gov/clinvar/), and OncoKB (Memorial Sloan Kettering Cancer Center; https://www.oncokb.org/). Literature searches were also conducted to identify pathogenic or likely pathogenic SNVs. Breast cancer driver genes were finally determined in compliance with the list in the report by Michailidow et al. [4].

***Estimation of structural variations and DNA copy number variants***

Large insertions, deletions, tandem duplications, and inversions with a tumor allele frequency of ≥0.07, depth threshold of ≥10, control depth of ≥10, and inversion size threshold of >1000 in the tumor were considered structural variation (SV) candidates.

Copy number variant (CNV) profiling was performed using DNAcopy version 1.56.0. (https://bioconductor.org/packages/release/bioc/html/DNAcopy.html), an R/Bioconductor package and Sclust [5]. Significantly amplified or deleted regions of the genome were identified using GISTIC 2.0 [6]. Tumor cellularity (purity) was analyzed using Sclust. To detect chromothripsis from WGS data, ShatterSeek v1.1 (https://github.com/parklab/ShatterSeek), an R package, was used based on previously established criteria [7]

***Analysis of cancer clonal evolution in LGASC and associated MSC***

MesKit version 1.6.0 (https://bioconductor.org/packages/release/bioc/html/MesKit.html), an R/Bioconductor-based package developed by Liu et al. [8], was used to analyze cancer clonal evolution based on WGS data. Sclust was used to estimate copy number, purity, and the CCF based on these values.

***Reverse transcription-polymerase chain reaction***

The existence of the *SMAD4::DCC* fusion transcript in LGASC and LNMSC was evaluated using reverse transcription-polymerase chain reaction (RT‒PCR). Briefly, total RNA was extracted from frozen LGASC and LNMSC tissues using TRIzol Reagent (Invitrogen, Waltham, MA) according to the manufacturer’s instructions. Total RNA was reverse transcribed to cDNA using SuperScript IV VILO Master Mix (Invitrogen) and subjected to PCR amplification using PrimeSTAR HS DNA polymerase (Takara, Kyoto, Japan). The forward PCR primer, with the nucleotide sequence 5'-CTACGAACGAGTTGTATCACC-3' (*SMAD4*-forward), targets exon 3 of *SMAD4;* the reverse primer, with the nucleotide sequence 5'-ACTTGAGTAGCACTGTGTCTC-3' (*DCC*-reverse), targets exon 3 of *DCC*. The amplified fragments that correspond to the predicted molecular size were subcloned into the pCR®︎-TOPO®︎ plasmid vector, and the nucleotide sequence was determined by Sanger sequencing using an ABI PRISM 3130xl Genetic Analyzer (Thermo-Fisher Scientific, Inc.).

***Western blotting***

To assess the existence of the *SMAD4::DCC* chimeric protein, western blotting was performed using the NuPAGE 4%–12% gradient Bis-Tris Protein Gel system (Thermo-Fisher Scientific, Inc.) with MOPS running buffer (Thermo-Fisher Scientific, Inc.). Proteins were extracted from frozen LGASC and LNMSC tissues using Cell Lysis Buffer (Cell Signaling Technology, Inc., Dancers, MA). We used the following antibodies to detect the *SMAD4::DCC* fusion protein: anti-SMAD4 rabbit monoclonal antibody (BLR133J, Bethyl Laboratories, Montgomery, TX, USA, 1:1,000) and anti-DCC mouse monoclonal antibody (A-1, Santa Cruz Biotechnology, Dallas, TX, USA, 1:1,000). Anti-vinculin (V9131, Sigma‒Aldrich, Merck KGaA, Darmstadt, Germany, 1:10,000) was used as a protein loading control. Secondary antibody reactions were performed using peroxidase-conjugated anti-mouse IgG (NA931, Cytiva, Tokyo, Japan, 1:100,000) or anti-rabbit IgG (NA934, Cytiva, 1:100,000). Detection was performed using the ImmunoStar LD-enhanced chemiluminescence detection reagent (FUJIFILM Wako Chemicals, Osaka, Japan).

**2. Supplementary figure and table legends**

**Fig. S1** GISTIC plots of copy number gain identified in the five metaplastic carcinomas of the breast samples. The size of each bar shows the G-score, which is the frequency multiplied by the average amplitude of the aberrations; q-values of more than 0.25 were considered significant. Twenty-nine genes were identified in the GISTIC peaks of copy number gain (8q24.3)

**Fig. S2** Estimated copy-number states of all chromosomes of Case 1 determined by Sclust. Brown and dark green lines show total clonal copy number and clonal minor allele copy number, respectively. Estimated tumor cellularity (purity) and ploidy based on Sclust analysis are shown on the left upper side of each figure. LGASC, low-grade adenosquamous carcinoma; LNMSC, lymph node metastasis consisting of high-grade metaplastic carcinoma of the breast with a predominant metaplastic squamous cell carcinoma component

**Table S1** Clinicopathological characteristics of three *de novo* high-grade metaplastic carcinomas

**Table S2** Whole-genome sequencing data (SNV, indel) of LGASC

**Table S3** Whole-genome sequencing data (SNV, indel) of LNMSC

**Table S4** Whole-genome sequencing data (SNV, indel) of M2T

**Table S5** Whole-genome sequencing data (SNV, indel) of M3T

**Table S6** Whole-genome sequencing data (SNV, indel) of M4T

**Table S7** Whole-genome sequencing data (SV) of LGASC

**Table S8** Whole-genome sequencing data (SV) of LNMSC

**Table S9** Whole-genome sequencing data (SV) of M2T

**Table S10** Whole-genome sequencing data (SV) of M3T

**Table S11** Whole-genome sequencing data (SV) of M4T

**References**

1. Reis-Filho J, Gobbi H, Reed AM, et al (2019) Metaplastic carcinoma. In: Allison K, et al (eds) World health organization breast tumours. Lyon, IARC, pp 134–138

2. Allison KH, Hammond MEH, Dowsett M et al (2020) Estrogen and progesterone receptor testing in breast cancer: ASCO/CAP guideline update. J Clin Oncol 38:1346-1366. https://doi.org/10.1200/jco.19.02309

3. Wolff AC, Hammond MEH, Allison KH et al (2018) Human epidermal growth factor receptor 2 testing in breast cancer: American society of clinical oncology/college of American pathologists clinical practice guideline focused update. Arch Pathol Lab Med 142:1364-1382. https://doi.org/10.5858/arpa.2018-0902-SA

4. Michailidou K, Lindström S, Dennis J et al (2017) Association analysis identifies 65 new breast cancer risk loci. Nature 551:92-94. https://doi.org/10.1038/nature24284

5. Cun Y, Yang TP, Achter V, Lang U, Peifer M (2018) Copy-number analysis and inference of subclonal populations in cancer genomes using Sclust. Nat Protoc 13:1488-1501. https://doi.org/10.1038/nprot.2018.033

6. Mermel CH, Schumacher SE, Hill B, Meyerson ML, Beroukhim R, Getz G (2011) GISTIC2.0 facilitates sensitive and confident localization of the targets of focal somatic copy-number alteration in human cancers. Genome Biol 12:R41. https://doi.org/10.1186/gb-2011-12-4-r41

7. Cortés-Ciriano I, Lee JJ, Xi R, Jain D, Jung YL, Yang L, Gordenin D, Klimczak LJ, Zhang CZ, Pellman DS, Park PJ (2020) Comprehensive analysis of chromothripsis in 2,658 human cancers using whole-genome sequencing. Nat Genet 52:331-341. https://doi.org/10.1038/s41588-019-0576-7

8. Liu M, Chen J, Wang X, Wang C, Zhang X, Xie Y, Zuo Z, Ren J, Zhao Q (2021) MesKit: A tool kit for dissecting cancer evolution of multi-region tumor biopsies through somatic alterations. Gigascience 10:giab036. https://doi.org/10.1093/gigascience/giab036
