# Supplementary figures and images for: Genetic analysis of low-grade adenosquamous carcinoma of the breast progressing to high-grade metaplastic carcinoma
Source: Breast Cancer Res Treat. 2023 Aug 31;202(3):563–73. doi: 10.1007/s10549-023-07078-9 (PMC10564816; doi:10.1007/s10549-023-07078-9)

Fig. S1

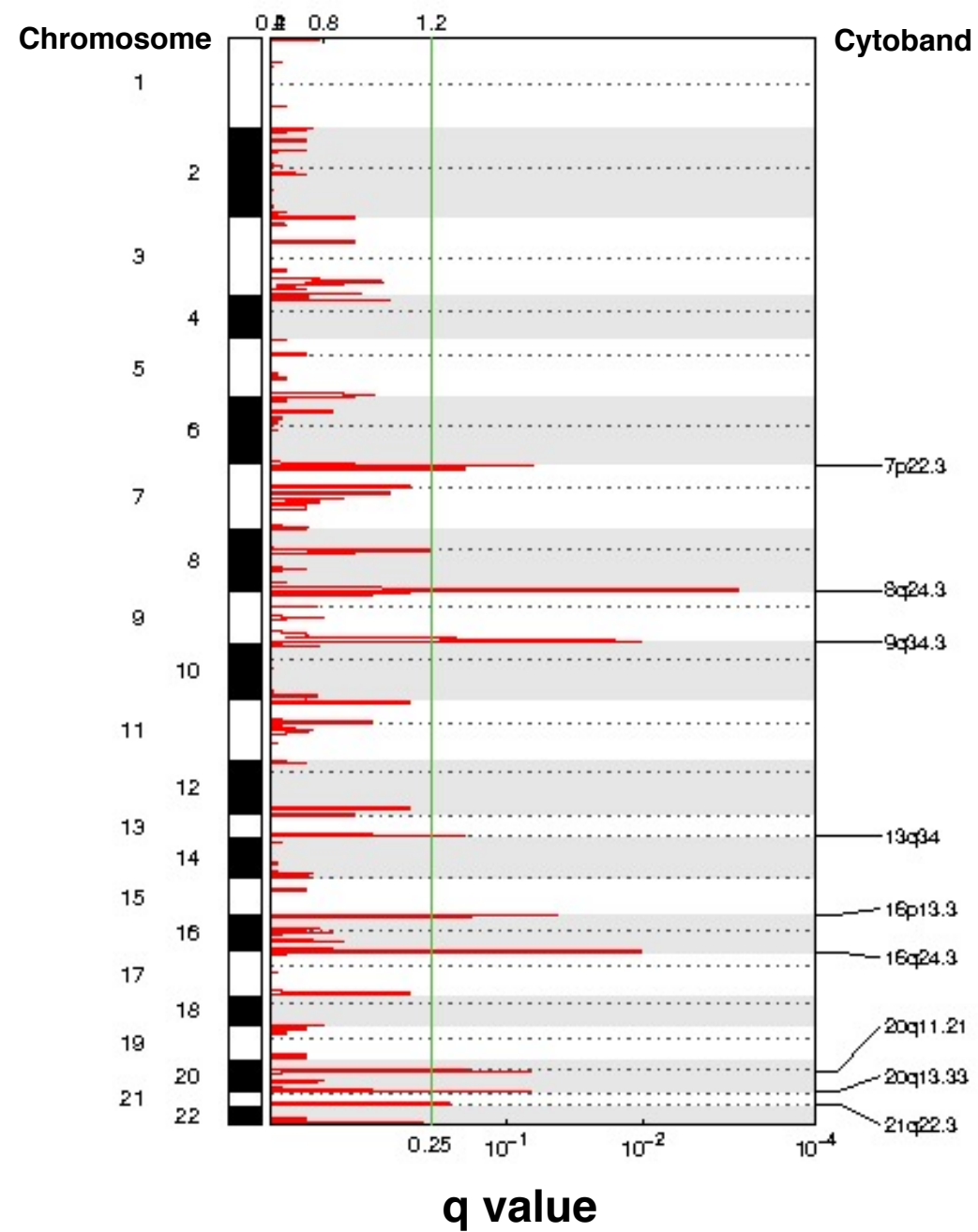

Fig. S2

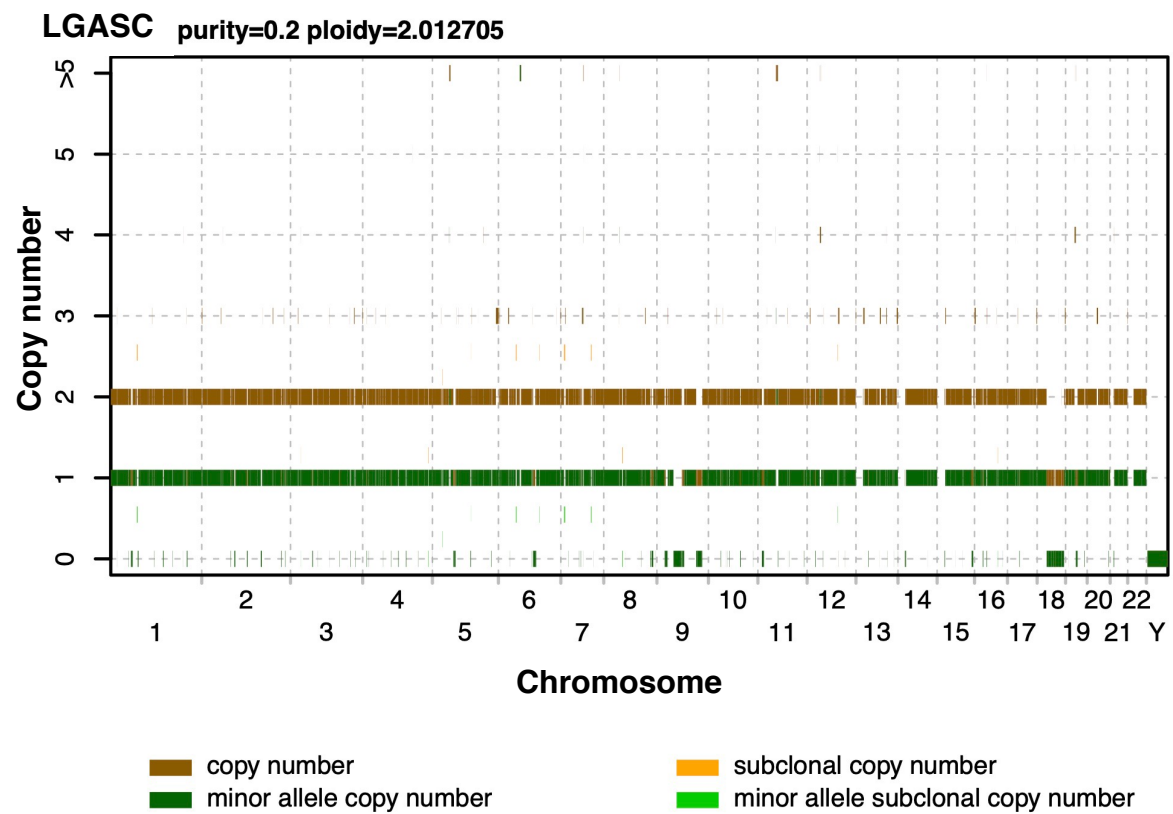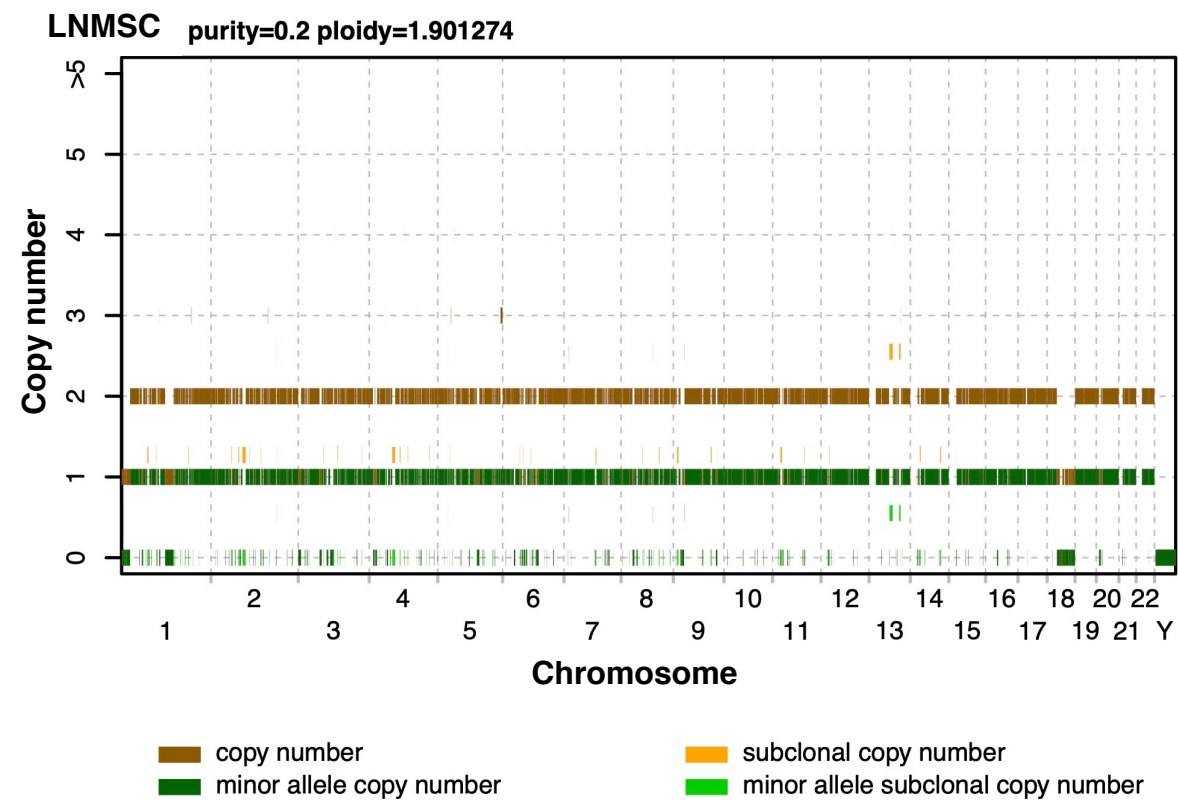

Supplement: Supplementary file 2 — (PDF 898 KB) [file 10549_2023_7078_MOESM2_ESM.pdf]
